# The Spectrum of Small Intestinal Lesions in Patients with Unexplained Iron Deficiency Anemia Detected by Video Capsule Endoscopy

**DOI:** 10.3390/medicina55030059

**Published:** 2019-02-27

**Authors:** Antonella Contaldo, Giuseppe Losurdo, Francesca Albano, Andrea Iannone, Michele Barone, Enzo Ierardi, Alfredo Di Leo, Mariabeatrice Principi

**Affiliations:** Section of Gastroenterology, Department of Emergency and Organ Transplantation, University of Bari, Piazza Giulio Cesare 11, 70124 Bari, Italy; contaldoantonella@gmail.com (A.C.); giuseppelos@alice.it (G.L.); albano.francesca@alice.it (F.A.); ianan@hotmail.it (A.I.); michele.barone@uniba.it (M.B.); ierardi.enzo@gmail.com (E.I.); alfredo.dileo@uniba.it (A.D.L.)

**Keywords:** anemia, videocapsule endoscopy, obscure occult bleeding, NSAIDs, PPIs

## Abstract

*Background and objectives*: Video-capsule endoscopy (VCE) has shown a large range (38–83%) of diagnostic yield in unexplained iron deficiency anemia (IDA) and obscure-occult bleeding. Therefore, we retrospectively investigated the VCE-detected spectrum and the prevalence of small bowel injuries and associated risk factors in inpatients with both of the above reported conditions. *Methods*: We selected inpatients with IDA (hemoglobin <12 g/dL in women, <13 g/dL in men) and obscure-occult bleeding. We excluded VCE indications other than IDA. Complete medical histories and laboratory tests were collected. All subjects underwent PillCam SB2/SB3. The VCE feature Lewis score was calculated when appropriate. We used the *t*-test and Fisher’s exact test for continuous and categorical variables, respectively, in univariate analysis. For multivariate analysis, we used binomial logistic regression. *Results*: We retrieved 109 patients (female:male ratio of 53:56; age 63.4 ± 18.9 years). Eighty patients (73.4%) showed ≥1 small bowel lesions. The Lewis score was calculated in 41 patients: 13 (31.7%) showed a mild (<135) and 28 (68.3%) a moderate-severe (135–790 and >790, respectively) score. In univariate analysis, the small bowel transit time (6.2 ± 2.9 versus 5.2 ± 2.1 h; *p* = 0.049) and non-steroidal anti-inflammatory drug use for at least two weeks (17.5% versus 0%; *p* = 0.01) were significantly higher in subjects with injuries. These associations were not confirmed at multivariate analysis. The severity of a lesion directly correlated with proton pump inhibitor (PPI) use and duration (not confirmed in multivariate analysis). VCE can reveal the source of obscure-occult bleeding in a high percentage of unexplained IDAs. A wide spectrum of endoscopic pictures may be found. Known as well as supposed risk factors for small bowel lesions may be detected.

## 1. Introduction

Iron deficiency anemia (IDA) may affect 1–2% of adults in Western countries [1]. According to the World Health Organization (WHO), anemia is defined as a hemoglobin concentration below 13 g/dL in men and 12 g/dL in non-pregnant women aged more than 15 years [2]. Ferritin is a marker of iron general status.Ferritin levels <30 µg/L without an inflammatory condition (normal C reactive protein levels) are consistent with iron deficiency. If inflammation is present, a ferritin value between 30 and 100 µg/L is suggestive of aninadequate amount of iron [3,4]. There are many causes of IDA; blood loss from the gastrointestinal tract is the most common in adults and postmenopausal women. For this reason, up to 13% of subjects with IDA are referred to gastroenterology units [5,6].

Obscure gastrointestinal hemorrhage is defined as persisting or recurring bleeding of unknown origin. In detail, obscure-occult bleeding is characterized by recurrent or persistent IDA and fecal occult blood test (FOBT) positivity. On the other hand, obscure-overt hemorrhage is defined by visible bleeding (i.e., melena and/or hematochezia). In both cases, gastroscopy and colonoscopy are unable to identify the source of the bleeding [7,8,9,10].

The literature does not provide a clear approach for IDA patients, although the British Society of Gastroenterology recommends a complete gastrointestinal investigation for anemia at all times [5,9]. Indeed, the cause of IDA remains unexplained in up to 30% of patients after upper/lower endoscopy [10] and small bowel blood loss represents 5–10% of all gastrointestinal bleeding [7,9]. Therefore, the European Society of Gastrointestinal Endoscopy (ESGE) guidelines advocate performing videocapsule endoscopy (VCE) as a first line examination for obscure gastrointestinal bleeding [11]. Despite the fact that the investigation may explore the entire small bowel in 79–90% of cases, the gaps in the literature on the range of the VCE diagnostic yield (38–83%) in obscure gastrointestinal bleeding need to be emphasized [7,12].

Based on this diagnostic yield discrepancy, the primary aim of this retrospective study was to depict both the prevalence and the spectrum of small bowel injury features detected by VCE in a cohort of inpatients with IDA and obscure-occult small bowel bleeding. The secondary aim was to explore potential predictive factors related to both the presence/absence and the severity of lesions detected by VCE.

## 2. Materials and Methods

### 2.1. Planning of the Study

This was a single-center observational retrospective study on inpatients with IDA, according to WHO classification [2], who were referred to a tertiary gastroenterology unit from December 2010 to December 2016. Our cohort included only admitted patients because the local setting of University Hospital Health Public only provided VCE procedures for inpatients affected by obscure-occult gastrointestinal bleeding.

All patients had displayed negative ileocolonoscopy and esophagogastroduodenoscopy and a positive fecal occult blood test (FOBT).

The exclusion criteria were: Overt gastrointestinal hemorrhage, menorrhagia or any overt source of extra-intestinal bleeding, inflammatory bowel diseases, celiac disease, chronic liver diseases, and inherited polyposis syndromes.

The study was performed in agreement with the Declaration of Helsinki. Being a retrospective study, which did not need investigations other than what was required for the clinical management of IDA and obscure-occult bleeding, the study was reviewed and approved after a meeting of the authors, all affiliated with the Gastroenterology Unit of Bari University Hospital Policlinico (Italy).

Written informed consent to undergo a VCE procedure (in the Italian language) was obtained from each patient. In the text it wasclearly reported: “I accept that demographic and clinical data and the outcome of the procedure can be managed anonymously for scientific purposes”. A blank copy of the informed consent sheet is attached as supplementary material and the mentioned sentence is highlighted.

### 2.2. Video Capsule Endoscopy Procedure

Before the investigation, complete medical histories (including medication and comorbidities) and laboratory tests were collected. Each patient’s medical history was collected by a direct interview performed by a physician at the time of admission. Medical histories as well as the results of laboratory and instrumental investigations were recorded in institutional medical archives.

Video capsule examinations were performed with Pillcam SB2 and SB3 (Medtronic, Dublin, Ireland). Informed consent was obtained from all patients. Capsule endoscopies were carried out after a 12 h fasting period. Polyethylene glycol (PEG; 3 L in the evening before and 1 L on the morning of the procedure) was administered according to ESGE guidelines in order to enhance the diagnostic yield [13], as confirmed by a recent meta-analysis [14]. Fluid and light meals were respectively allowed 2 and 4 h after capsule swallowing. The data recorder was removed after 12 h of registration. The recorded digital information was downloaded and analyzed using Rapid software.

Two endoscopists, who were blind to each patient’s history (A.C.; M.P.) and experienced in VCE (more than 100 previous examinations), reviewed the videos. The capsule endoscopy findings were assessed. The small bowel area was divided into three tertiles (proximal, middle, and distal) based on recording time duration [15]. Lesions were defined as follows: (a) “petechia”: circular area of crimson mucosa with preservation of villi; (b) “denuded area”: loss of villous architecture without clear breach of the epithelium; (c) “angiodysplasia”: enlarged blood vessels, usually a consequence of artero-venous malformations [16]. Other recorded alterations were: Mucosal breaks (i.e., ulcers and erosion), hemorrhagic areas, strictures, and neoplasms. The Lewis score was estimated where indicated [15]. This score characterizes small bowel mucosal inflammatory changes by evaluating three parameters: Villous edema, ulcer, and stenosis. A score of <135 describes a normal/clinically insignificant picture or a mild inflammation, while a score between 135 and 790, a moderate, and a score >790, a severe inflammation. Due to the characteristics of the Lewis score, its application was considered inappropriate for angiodysplasiae and cancers; therefore, in such cases it was not calculated.

### 2.3. Statistical Analysis

Continuous variables were expressed as mean ± standard deviation, whereas the categorical ones were expressed as percentages.

For univariate analysis, comparison between continuous variables was performed using Student’s *t*-test, while the Chi-squared test without Yates’s correction was used for categorical variables. This analysis was applied in order to compare patients with lesions at VCE topatients without lesions. Variables with *p* ≤ 0.10 at univariate analysis were analyzed by multivariate analysis using binomial logistic regression [17]. Multivariate analysis aimed to establish factors independently associated to: (i) the presence of lesions at VCE; and, (ii) the severity of lesions according to Lewis score. In this last analysis patients with moderate and severe Lewis scores were merged in order to avoid the dispersion of data. Odd ratios (OR) and 95% confidence intervals (CI) were calculated as estimations of the risk. The variable was inversely associated to the presence of lesions when 0 < OR < 1; conversely, it was directly correlated when OR > 1.

All statistical tests were two-tailed. The analysis was performed using the software SPSS Statistics for Windows, Version 23.0. (IBM Corp., Armonk, NY, USA).

## 3. Results

### 3.1. Patients

We enrolled 109 patients; the mean age was 63 ± 18.9 years, with an age range of 20–95, and the male:female ratio was 53:56. Of the patients, 43 were affected by arterial hypertension, 16 by chronic kidney disease, 6 by obesity, and 5 by joint disorders. At enrolment, 25 patients were using acetylsalicylate (aspirin) as an antiplatelet aggregator, 42 were using other non-steroidal anti-inflammatory drugs (NSAIDs), 31 proton pump inhibitors (PPI), and 18 oral anticoagulants. NSAIDs were used by 14 patients for symptomatic therapy of osteo-articular pain, prescribed by an orthopedics or rheumatology specialist, 5 for connectivitis, and 23 as self-medication. PPIs were used by 8 patients in order to prevent gastric damage due to aspirin or NSAIDs, and the remaining 23 for gastro-esophageal reflux disease with (7 subjects) or without (10 subjects) erosive esophagitis and reflux-like dyspepsia (6 subjects). The demographic and clinical characteristics of our study population are shown in Table 1.

### 3.2. Small Bowel Capsule Endoscopy Findings

The mean transit time evaluated by VCE recording in all patients was 5.9 ± 2.7 h.

**Figure 1:**
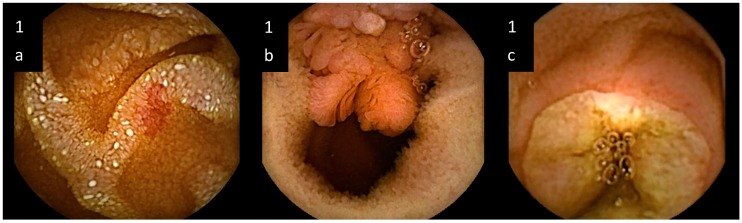
Samples of small bowel lesions detected by video capsule endoscopy in unexplained iron deficiency anemia. (**a**) artero-venous malformation, i.e., “angiodysplasia”, characterized by enlarged blood vessels; (**b**) neoplasm protruding into the lumen; (**c**) ulcer, characterized by a mucosal break.

Out of 109 patients, 80 (73.4%) showed VCE pathological pictures; while in the other 29 (26.6%), a normal finding was observed. The 80 patients with VCE abnormalities showed an overall number of 116 lesions. Indeed, we identified 14 out of 80 patients (17.5%) with multiple lesions, whose peculiarities are summarized in Table 2. Pathological findings showedpetechiae (11 out of 80 patients: 13.7%), denuded areas (3 out of 80: 3.75%), mucosal breaks like erosions or ulcers (29 out of 80: 36.2%), hemorrhagic areas (7 out of 80: 8.75%), angiodysplasiae (25 out of 80: 31.25%), strictures (5 out of 80: 6.25%) and neoplasms (15 out of 80: 18.75%). Some samples of the spectrum of observed lesions are reported in Figure 1.

The site of lesions was the proximal tertile in 49 out of 80 (61.2%) cases, the second tertile in 26 out of 80 (32.5%), and the distal tertile in 41 out of 80 (51.2%). For 36 out of 80 (45.0%) patients, the same findings were simultaneously allocated at different tertiles.

Out of 80 patients, 41 showed an inflammatory pattern and, therefore, were graded according to Lewis score. In detail, 13 out of 41 patients (31.7%) showed a mild and 28 out of 41 (68.3%) a moderate-severe score.

### 3.3. Univariate and Multivariate Analyses

As indicated above, univariate and multivariate analyses assessed predictive factors related to both the presence or absence and the severity of lesions.

#### 3.3.1. Presence or Absence of Lesions

Univariate analysis demonstrated that a longer small bowel transit time and the use of NSAIDs for at least two weeks were significantly associated with the presence of lesions (OR = 1.13; 95% CI 1.02–1.31; *p* = 0.049 and OR = 12.86; 95% CI 0.74–223.1; *p* = 0.01, respectively). The use of oral anticoagulants demonstrated a trend in the association with lesions at VCE, despite a not statistical significance (OR = 3.38; 95% CI 0.73–15.70; *p* = 0.10). No correlation was observed for the other considered parameters, which are summarized in Table 3.

Multivariate analysis (Table 4) did not confirm any significance observed inunivariate tests. Indeed, neither of these factors were associated with the presence of VCE injuries: Small bowel transit time (OR = 0.86; 95% CI 0.72–1.03; *p* = 0.09) and NSAID usefor at least 2 weeks (OR = 2.15; 95% CI 0.44–10.50; *p* = 0.34). Similarly, oral anticoagulant administration did not show any significance (OR = 2.50; 95% CI 0.46–4.56; *p* = 0.36).

Of relevance, all eight patients using both NSAIDs or aspirin and PPI showed small bowel lesions.

#### 3.3.2. Severity of Lesions

This analysis involved only 41 patients, i.e., those showing inflammatory lesions at VCE. As previously mentioned, patients with moderate and severe Lewis scores were merged into a single group. When patients with a mild score made up the reference group, a high Lewis score correlated to PPI use (OR = 12.0; 95% CI 1.4–100; *p* = 0.01) and duration of use (OR = 21; 95% CI 1.2–792; *p* = 0.03) at univariate analysis. However, at multivariate analysis, no factor was statistically associated to the Lewis score.

## 4. Discussion

The results of the present study are in agreement with those of the studies describing the high diagnostic suitability of VCE for the detection of sources of small bowel bleeding in IDA patients [7,8,9,10]. Moreover, they are very close to what was reported by Koulaouzidis et al. in a systematic review estimating a pooled diagnostic ability of 66.6% (95% CI: 61–72.3%) [18]. Therefore, it is possible that gaps in the VCE performance literature depend on the adequacy of the selection of IDA patients to be investigated [10,19]. A further explanation of our results may be the investigation’s optimal timing (i.e., within three days of admission) [20]. On the other hand, literature data show that the state of the inpatient or outpatient does not affect VCE performance. Indeed, our cohort included only admitted patients because the local setting of University Hospital Health Public System only provides VCE procedures for inpatients affected by obscure-occult gastrointestinal bleeding. As expected, our results confirm that an appropriate skilled approach to improving the VCE diagnostic yield should be directed at IDA patients with positive FOBT and negative bidirectional endoscopy. Additionally, this strategy should take into account exclusion criteria, such as the presence of IDA-inducing gastrointestinal conditions (atrophic gastritis or malabsorption) and a detailed medical history excluding extra-intestinal disorders accounting for IDA. Despite the fact that the above-mentioned cautions allow a high VCE diagnostic power to be obtained, some limitations of this investigation could explain the small percentage of failures in identifying the cause of IDA. They may essentially be represented by “missed lesions” and an inability to precisely locate injuries in small bowel districts [10,19,21].

In this study, we showed that a wide spectrum of VCE pictures may be found in IDA patients. This finding could suggest the need to promote morphologic agreements in discriminating and describing the different pictures, which may presumably account for the large VCE range (38–83%) of the diagnostic yield in IDA [7,12]. As a result, in our analysis, the majority of detected sources were vascular (11 petechiae and 25 angiodysplasiae) and inflammatory (29 mucosal breaks like erosions or ulcers and 5 strictures). Moreover, in this study, vascular lesions were frequently observed in the proximal small bowel even though the retrospective type of study did not allow a comparison of VCE with enteroscopy. However, despite the fact that the reason for this location is unclear, this finding could explain what is reported in the literature, i.e., the good performance of enteroscopy in identifying and, mostly, in treating this type of injury [22,23]. Finally, a recent European study showed that the diagnostic yield of VCE was not adequately effective in the case of small bowel masses when they did not protrude into the lumen [24]. This potential limit [25] was in agreement with our study, which detected 15 (12.9%) single neoplasms.

Univariate analysis, directed at identifying risk factors associated with the presence or absence of small bowel injury, showed that slow intestinal transit and NSAID use directly correlated with the possibility of finding injuries with VCE. Concerningthe first possible risk factor identified by this study, Hejazi et al. have shown that VCE could potentially be used for measuring small bowel transit time, even though they reported that it does correlate with undefined IDA-related intestinal lesions [26]. Therefore, the disagreement between our study and that of Hejazi et al. could encourage further investigations focused on this topic. Conversely, the correlation between NSAID use and small bowel lesions was expected, and the absence of significance at multivariate analysis is likely due to the small number of patients included in the analysis and the high number of considered variables. Indeed, many studies have identified different potential pathogenic mechanisms of NSAID-induced enteropathy [27,28,29,30]. In this context, the manipulation of the microbiota seems to be a promising approach in NSAID intestinal damage prevention [30,31,32,33]. Equally, treatments with Bifidobacterium, Lactobacilli, and *Faecalibacteriaum prausnitzii* have shown similar promising results in animal models [30].

In our study, the risk factors associated with the severity of small bowel injury at univariate analysis demonstrated a positive association with PPI use and duration, which was not confirmed by multivariate analysis presumably because of the small sample size. While a possible mechanistic explanation of damage may be evident for the 8patients usingPPI in association with NSAIDs or aspirin, it is unclear for patients using PPIs alone, since their association with small bowel injury has not been well explored in the literature and is controversial [34]. In a recent case-control study, there was a significant increase in the risk of lower gastrointestinal bleeding due to NSAIDs or aspirin with concomitant PPI use [35]. Interestingly, a randomized clinical trial was performed on healthy volunteers allocated to receive celecoxib plus placebo or rabeprazole. In the PPI group, the authors observed an increased rate of small bowel injury (44.7% versus 16.7%) [36]. Despite the fact that these observations have not been clearly explained, it has been hypothesized that a relative (but not absolute) increase in small bowel hemorrhage in PPI users may be due to low ascorbic acid assimilation or promotion of bleeding by pre-existing lesion [37]. Finally, some authors suggested that acid-gastric suppression induced by PPI is associated with changes in small bowel microbiota [38]. We can assume that, in addition to bile acids [39], bacterial mucosal translocation could trigger an inflammatory cascade with consequent intestinal damage. Nevertheless, further studies are mandatory in order to clarify the possibility of an association between PPIs and intestinal lesions in the absence of simultaneous NSAID use.

## 5. Conclusions

In conclusion, our study highlighted the convincing usefulness of VCE in providing clear information in the complex scenario of unexplained IDA by detailing the wide spectrum of VCE detectable lesions. Moreover, the possibility of risk factors for intestinal injuries were outlined in our analysis, while a clearer definition of accurate predictors of the presence and severity of small bowel IDA-related lesions is warranted in prospective studies.

## Figures and Tables

**Table 1:** Baseline demographic and clinical characteristics of the 109 enrolled patients.

Age, Years (Mean ± Standard Deviation)	63.4 ± 18.9
Female/male sex ratio	53/56
Hemoglobin (g/dL), mean ± standard deviation	10.1 ± 1.2
NSAIDs, *n* (%)	31 (28.4)
NSAIDs assumption for at least 2 weeks, *n* (%)	14 (12.8)
Aspirin, *n* (%)	25 (22.9)
Oral anticoagulants, *n* (%)	18 (16.5)
Probiotics, *n* (%)	14 (12.8)
Antibiotics, *n* (%)	13 (11.9)
PPI, *n* (%)	42 (38.5)
PPI assumption, *n* (%) <2 weeks 2 weeks–3 months >3 months	7 (6.4) 21 (19.3) 14 (12.8)
NSAIDs + Oral anticoagulants, *n* (%)	2 (1.8)
NSAIDs + Oral anticoagulants + PPI, *n* (%)	3 (2.7)
NSAIDs + PPI, *n* (%)	6 (5.5)
NSAIDs + aspirin + PPI, *n* (%)	2 (1.8)
Weight loss, *n* (%)	45 (41.3)
Occlusive symptoms, *n* (%)	2 (1.8)
Diabetes, *n* (%)	28 (25.7)
Hypertension, *n* (%)	43 (39.4)
Chronic kidney disease, *n* (%)	16 (14.7)
Obesity, *n* (%)	6 (5.5)
Arthrosis/arthritis, *n* (%)	5 (4.6)

NSAIDs: non-steroidal anti-inflammatory drugs; PPI: proton pump inhibitor.

**Table 2:** Patients (*n* = 14) with multiple lesions at video capsule endoscopy (VCE).

Association of Lesions Detected at VCE	Number of Patients
Strictures + mucosal breaks	5
Petechiae + mucosal breaks	2
Petechiae + hemorrhagic areas	2
Petechiae + angiodysplasiae	2
Angiodysplasiae + hemorrhagic areas	1
Neoplasms + hemorrhagic areas	1
Petechiae + mucosal breaks + denuded areas	1

**Table 3:** Univariate analysis comparing clinical and demographic features of patients with and without lesions at VCE.

	Presence of Lesions at VCE (*n* = 80)	Absence of Lesions at VCE (*n* = 29)	*p* Value
Age (mean ± standard deviation)	65 ± 17.7	59.0 ± 21.5	0.14
Small bowel transit time (hours, mean ± standard deviation)	6.2 ± 2.9	5.2 ± 2.1	**0.049**
Female sex, *n* (%)	38 (47.5)	15 (51.7)	0.83
Hemoglobin (g/dL), mean ± standard deviation	9.9 ± 1.3	10.1 ± 1.3	0.42
NSAIDs, *n* (%)	25 (31.2)	6 (20.7)	0.34
NSAIDs assumption for at least 2 weeks, *n* (%)	14 (17.5)	0 (0)	**0.01**
Aspirin, *n* (%)	18 (22.5)	7 (24.1)	0.52
Oral anticoagulants, *n* (%)	16 (20)	2 (6.9)	0.10
Probiotics, *n* (%)	10 (12.5)	4 (13.8)	0.86
Antibiotics, *n* (%)	11 (13.8)	2 (6.9)	0.33
PPI, *n* (%)	30 (37.5)	12 (41.4)	0.71
PPI assumption, *n* (%) <2 weeks 2 weeks–3 months >3 months	4 (5) 15 (18.8) 11 (13.8)	3 (10.3) 6 (20.6) 3 (10.3)	0.74
NSAIDs + Oral anticoagulants, *n* (%)	2 (2.5)	0 (0)	0.39
NSAIDs + Oral anticoagulants + PPI, *n* (%)	3 (3.8)	0 (0)	0.29
NSAIDs + PPI, *n* (%)	6 (7.5)	0 (0)	0.13
NSAIDs + aspirin + PPI, *n* (%)	2 (2.5)	0 (0)	0.39
Manifest bleeding, *n* (%)	30 (37.5)	11 (37.9)	0.96
Occlusive symptoms, *n* (%)	2 (2.5)	0 (0)	0.39
Diabetes, *n* (%)	22 (27.5)	6 (20.7)	0.47
Hypertension, *n* (%)	32 (40)	11 (37.9)	0.84
Chronic kidney disease, *n* (%)	12 (15)	4 (13.8)	0.87
Obesity, *n* (%)	6 (7.5)	0(0)	0.13
Arthrosis/arthritis, *n* (%)	3 (3.8)	2 (6.9)	0.49

NSAIDs: non-steroidal anti-inflammatory drugs; PPI: proton pump inhibitor; VCE: video-capsule endoscopy.

**Table 4:** Estimations of risk, expressed as odd ratios (OR), for the presence of lesions detected at VCE, both in univariate and multivariate analyses (binomial logistic regression).

	Univariate Analysis		Multivariate Analysis	
	OR (95% Confidence Interval)	*p* Value	OR (95% Confidence Interval)	*p* Value
Small bowel transit time	1.13 (1.02–1.31)	0.049	0.86 (0.72–1.03)	0.09
NSAID assumption for at least 2 weeks	12.86 (0.74–223.1)	0.01	2.15 (0.44–10.50)	0.34
Oral anticoagulants	3.38 (0.73–15.70)	0.10	2.50 (0.46–4.56)	0.36

NSAIDs: non-steroidal anti-inflammatory drugs.

## References

[1] Dahlerup J.F., Eivindson M., Jacobsen B.A., Jensen N.M., Jorgensen S.P., Laursen S.B. (2015). Diagnosis and treatment of unexplained anemia with iron deficiency without overt bleeding. Dan. Med. J..

[2] World Health Organisation (2008). Worldwide Prevalence of Anaemia 1993–2005.

[3] Hagve T.A., Lilleholt K., Svendsen M. (2013). Iron deficiency anaemia interpretation of biochemical and haematological findings. Tidsskr. Nor. Laegeforen.

[4] Reinisch W., Staun M., Bhandari S., Muñoz M. (2013). State of the iron: How to diagnose and efficiently treat iron deficiency anemia in inflammatory bowel disease. J. Crohns Colitis.

[5] Goddard A.F., James M.W., McIntyre A.S., Scott B.B. (2011). Guidelines for the management of iron deficiency anaemia. Gut.

[6] Holleran G.E., Barry S.A., Thornton O.J., Dobson M.J., McNamara D.A. (2013). The use of small bowel capsule endoscopy in iron deficiency anaemia: Low impact on outcome in the medium term despite high diagnostic yield. Eur. J. Gastroenterol. Hepatol..

[7] Gerson L.B., Fidler J.L., Cave D.R., Leighton J.A. (2015). ACG Clinical guideline: Diagnosis and management of small bowel bleeding. Am. J. Gastroenterol..

[8] Viazis N., Anastasiou J., Karamanolis D.G. (2016). Small bowel capsule endoscopy for the investigation of obscure gastrointestinal bleeding: When we should do it and what should we expect. Acta Gastroenterol. Belg..

[9] Milano A., Balatsinou C., Filippone A., Caldarella M.P., Laterza F., Lapenna D., Pierdomenico S.D., Pace F., Cuccurullo F., Neri M. (2011). A prospective evaluation of iron deficiency anemia in the GI endoscopy setting: Role of standard endoscopy, videocapsule endoscopy, and CT-enteroclysis. Gastrointest. Endosc..

[10] Naut E. (2016). The approach to occult gastrointestinal bleed. Med. Clin. N. Am..

[11] Pennazio M., Spada C., Eliakim R., Keuchel M., May A., Mulder C.J., Rondonotti E., Adler S.N., Albert J., Baltes P. (2015). Small-bowel capsule endoscopy and device-assisted enteroscopy for diagnosis and treatment of small-bowel disorders: European Society of Gatrointestinal Endoscopy (ESGE) Clinical Guidelines. Endoscopy.

[12] Rondonotti E., Villa F., Mulder C.J., Jacobs M.A., de Franchis R. (2007). Small bowel capsule endoscopy in 2007: Indications, risks and limitations. World J. Gastroenterol..

[13] Gunjan D., Sharma V., Rana S.S., Bhasin D.K. (2014). Small bowel bleeding: A comprehensive review. Gastroenterol. Rep..

[14] Rokkas T., Papaxoinis K., Triantafyllou K., Pistiolas D., Ladas S.D. (2009). Does purgative preparation influence the diagnostic yield of small bowel video capsule endoscopy? A meta-analysis. Am. J. Gastroenterol..

[15] Gralnek I.M., Defranchis R., Seidman E., Leighton J.A., Legnani P., Lewis B.S. (2008). Developmentof a capsule endoscopy scoring index for small bowel mucosal inflammatory change. Aliment. Pharmacol. Ther..

[16] Ingrosso M., Sabba C., Pisani A., Principi M., Gallitelli M., Cirulli A., Francavilla A. (2004). Evidence of small-bowel involvement in hereditary hemorrhagic telangiectasia: A capsule-endoscopic study. Endoscopy.

[17] Onali S., Kalafateli M., Majumdar A., Westbrook R., O’Beirne J., Leandro G., Patch D., Tsochatzis E.A. (2017). Non-selective beta-blockers are not associated with increased mortality in cirrhotic patients with ascites. Liver Int..

[18] Koulaouzidis A., Rondonotti E., Giannakou A., Plevris J.N. (2012). Diagnostic yield of small bowel capsule endoscopy in patients with iron deficiency anemia: A systematic review. Gastrointest. Endosc..

[19] Muhammad A., Vidyarthi G., Brady P. (2014). Role of small bowel capsule endoscopy in the diagnosis and management of iron deficiency anemia in elderly: A comprehensive review of the current literature. World. J. Gastroenterol..

[20] Singh A., Marshall C., Chaudhuri B., Okoli C., Foley A., Person S.D., Bhattacharya K., Cave D.R. (2013). Timing of video capsule endoscopy relative to overt obscure GI bleeding: Implications from a retrospective study. Gastrointest. Endosc..

[21] Lewis B.S., Eisen G.M., Friedman S. (2005). A pooled analysis to evaluate results of capsule endoscopy trials. Endoscopy.

[22] Plotkin E., Imaeda A. (2016). Small intestinal angioectasias are not randomly distributed in small bowel and most may be reached by push enteroscopy. J. Clin. Gastroenterol..

[23] Nennstiel S., Machanek A., von Delius S., Neu B., Haller B., Abdelhafez M., Schmid R.M., Schlag C. (2017). Predictors and characteristics of angioectasias in patients with obscure gastrointestinal bleeding identified by video capsule endoscopy. United Eur. Gastroenterol. J..

[24] Rondonotti E., Pennazio M., Toth E., Menchen P., Riccioni M.E., De Palma G.D., Scotto F., De Looze D., Pachofsky T., Tacheci I. (2008). Small bowel neoplasms in patients undergoing video capsule endoscopy: A multicenter European study. Endoscopy.

[25] Annibale B., Capurso G., Baccini F., Lahner E., D’Ambra G., Di Giulio E., DelleFave G. (2003). Role of small bowel investigation in iron deficiency anemia after negative endoscopic/histologic evaluation of the upper and lower gastrointestinal tract. Dig. Liver Dis..

[26] Hejazi R.A., Bashashati M., Saadi M., Mulla Z.D., Sarosiek I., McCallum R.W., Zuckerman M.J. (2016). Video capsule endoscopy: A tool for assessment of small bowel transit time. Front. Med..

[27] Takeuchi K., Sathon H. (2015). NSAID-Induced small intestinal damage-Roles of various pathogenic factors. Digestion.

[28] Blackler R.W., Gemici B., Manko A., Wallace J.L. (2014). NSAID-gastroenteropathy: New aspects of pathogenesis and prevention. Curr. Opin. Pharmacol..

[29] Marlicz W., Łoniewski I., Grimes D.S., Quigley E.M. (2014). Nonsteroidal Anti-Inflammatory drugs, proton pump inhibitors and gastrointestinal injury: Contrasting interactions in the stomach and small intestine. Mayo Clin. Proc..

[30] Syer S.D., Blackler R.W., Martin R., De Palma G., Rossi L., Verdu E., Bercik P., Surette M.G., Aucouturier A., Langella P. (2015). NSAID enteropathy and bacteria: A complicated relationship. J. Gastroenterol..

[31] Uejima M., Kinouchi T., Kataoka K., Hiraoka I., Ohnishi Y. (1996). Role of intestinal bacteria in ileal ulcer formation in rats treated with a nonsteroidal anti-inflammatory drug. Microbiol. Immunol..

[32] Bjarnason I., Hayllar J., Smethurst P., Price A., Gumpel M.J. (1992). Metronidazole reduce interstinal inflammation and blood loss in non-steroidal anti-inflammatory drug induced enteropathy. Gut.

[33] Montenegro L., Losurdo G., Licinio R., Zamparella M., Giorgio F., Ierardi E., Leo A.D., Principi M. (2014). Non steroidal anti-inflammatory drug induced damage on lower gastro-intestinal tract: Is there an involvement of microbiota?. Curr. Drug Saf..

[34] Lué A., Lanas A. (2016). Protons pump inhibitor treatment and lower gastrointestinal bleeding: Balancing risks and benefits. World J. Gastroenterol..

[35] Lanas Á., Carrera-Lasfuentes P., Arguedas Y., García S., Bujanda L., Calvet X., Ponce J., Perez-Aísa Á., Castro M., Muñoz M. (2015). Risk of upper and lower gastrointestinal bleeding in patients taking nonsteroidal anti-inflammatory drugs, antiplatelet agents, or anticoagulants. Clin. Gastroenterol. Hepatol..

[36] Arroyo R.C., Polo-Tomas M., Roncalés M.P., Scheiman J., Lanas Á. (2012). Lower GI bleeding is more common than upper among patients on dual antiplatelet therapy: Long-term follow-up of a cohort of patients commonly using PPI co-therapy. Heart.

[37] Lanas A., García-Rodríguez L.A., Polo-Tomás M., Ponce M., Quintero E., Perez-Aisa M.A., Gisbert J.P., Bujanda L., Castro M., Muñoz M. (2011). The changing face of hospitalisation due to gastrointestinal bleeding and perforation. Aliment. Pharmacol. Ther..

[38] Wallace J.L., Syer S., Denou E., de Palma G., Vong L., McKnight W., Jury J., Bolla M., Bercik P., Collins S.M. (2011). Pomp proton inhibitors exacerbate NSAID-induced small intestinal injury by inducing dysbiosis. Gastroenterology.

[39] Petruzzelli M., Vacca M., Moschetta A., Sasso R.C., Palasciano G., van Erpecum K.J., Portincasa P. (2007). Intestinal mucosal damage caused by non-steroidal anti-inflammatory drugs: Role of bile salts. Clin. Biochem..

